# High-velocity impact of solid objects on Non-Newtonian Fluids

**DOI:** 10.1038/s41598-018-37543-1

**Published:** 2019-02-04

**Authors:** Thijs C. de Goede, Karla G. de Bruin, Daniel Bonn

**Affiliations:** 1 Van der Waals-Zeeman Institute, Institute of Physics, University of Amsterdam, Science Park 904, 1098 XH Amsterdam, The Netherlands; 2 Netherlands Forensic Institute, Laan van Ypenburg 6, 2497 GB The Hague, The Netherlands

## Abstract

We investigate which property of non-Newtonian fluids determines the deceleration of a high-speed impacting object. Using high-speed camera footage, we measure the velocity decrease of a high-speed spherical object impacting a typical Newtonian fluid (water) as a reference and compare it with a shear thickening fluid (cornstarch) and a shear thinning viscoelastic fluid (a weakly cross-linked polymer gel). Three models describing the kinetic energy loss of the object are considered: fluid inertia, shear thickening and viscoelasticity. By fitting the three models to the experimental data, we conclude that the viscoelastic model works best for both the cornstarch and the polymer gel. Since the cornstarch is also viscoelastic, we conclude that the ability to stop objects of these complex fluids is given by their viscoelasticity rather than shear thickening or shear thinning.

## Introduction

The impact of solid objects on liquid surfaces has been studied intensively over the last century due to its relevance in the water entry of military projectiles^1,2^, the construction of naval vehicles^3,4^ and the motion of water-walking lizards^5^. Understanding the forces and mechanics for the kinetic energy dissipation is key for understanding the fluid behaviour during impact. For Newtonian fluids, the kinetic energy of the impacting object is dissipated by either viscous or inertial forces, depending on the impact velocity of the object^6^. However, it has been known for some time that the use of non-Newtonian fluids can provide efficient ways of changing the impact dynamics, which could have many useful applications. For instance, a recent study has shown that the object stopping power of Kevlar body vests can be significantly enhanced by impregnating the Kevlar layers with a suspension^7^, which is the foundation of liquid body armour designs^8–12^. These studies proposed that the shear thickening properties of the suspension, an increase in viscosity when subjected to a shear stress, is responsible for the enhanced kinetic energy dissipation in the Kevlar vests.

To describe such kinetic energy dissipation in a shear thickening fluid, Waitukaitis and Jaeger recently investigated relatively low velocity impacts (*v* ~ 1 m/s) on cornstarch dissolved in water^13^. They proposed that the kinetic energy dissipation inside a shear thickening fluid could be described using an ‘added mass’ model^14,15^. In the model, the shear thickening liquid underneath the object solidifies during impact due to the formation of so-called jamming clusters^16–19^, creating a solid ‘plug’ underneath the object. This plug is subsequently pushed downward by the object and causes a downward flow in the surrounding liquid, increasing the mass that the object has to displace over time. With this model, they were able to predict the deceleration of an object impacting in shear thickening fluids.

However, shear thickening might not be the only mechanism for describing the enhanced stopping power of suspensions. In practice, most suspensions are also viscoelastic and can therefore react elastically on an external stress, depending on the timescale^20^. Here, the characteristic timescale of the developing visco-elastic stress depends on the impact velocity of the object. The possibility exists that for the liquid body amour studies, where the impact velocities are two magnitudes higher compared to the study of Waitukaitis and Jaeger^13^ (*v* ~ 100 m/s), the mechanism could be different.

In this study, we compare how a high speed spherical plastic bullet is decelerated in three fluids: water, a purely shear thickening fluid and a viscoelastic shear thinning fluid. Using high-speed camera footage and three kinetic-energy dissipation models, we show that both the shear thickening and viscoelastic fluids respond viscoelastically to the object’s impact.

## Rheological Properties Fluids

**Figure 1:**
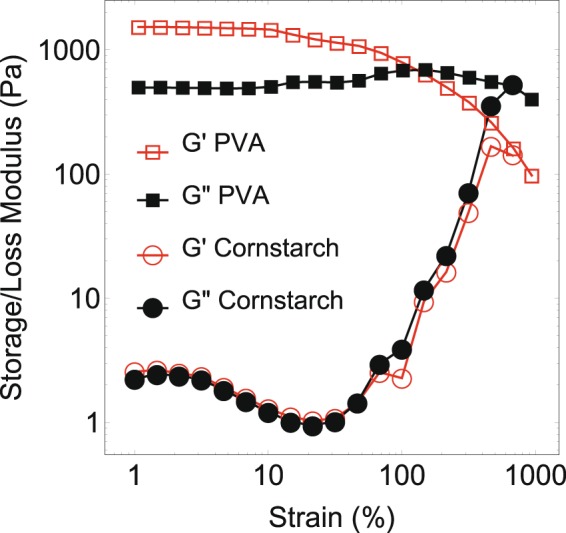
Storage (open red symbols) and loss modulus (solid black symbols) of PVA (squares) and cornstarch (circles) as a function of induced strain.

As typical shear thickening and viscoelastic fluids we selected cornstarch dissolved in water and a polyvinyl alcohol (PVA) borax solution, respectively. Borax (sodium tetraborate) acts as a weak crosslinker between the PVA polymers^21^. In order to assess the properties of these fluids, their storage (*G*′) and loss (*G*″) moduli were measured in a rheometer using a cone-plate geometry with an angular frequency *ω* of 10 rad/s (Fig. 1). An oscillatory shear experiment was used to avoid complications due to e.g. particle migration^22^. In this measurement, cornstarch was carefully density matched using CsCl to avoid the influence of particle sedimentation^22–24^. The results show that for PVA, both *G*′ and *G*″ stay relatively constant as a function of the applied deformation, after which they both decrease when some critical strain is exceeded. For cornstarch the opposite effect can be seen, with both moduli increasing strongly when the strain exceeds a critical value. This confirms that PVA is a shear thinning and cornstarch a shear thickening fluid.

## Bullet Impact Measurements

**Figure 2:**
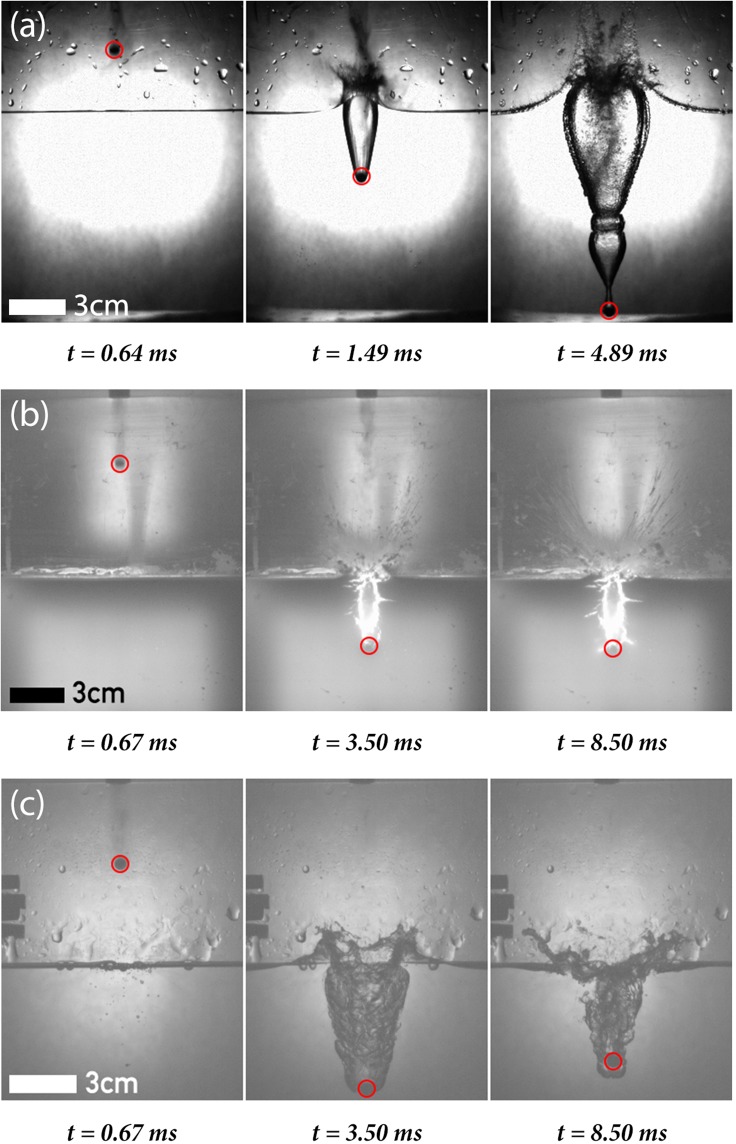
High-speed images of a plastic object (inside the red circles) penetrating water (**a**), cornstarch (**b**) and PVA (**c**) in the small container. Here, *t* = 0 is the moment that the object appears in frame.

We subsequently measured the temporal decrease in velocity of a high-speed object in water, cornstarch and PVA using high-speed camera footage (Fig. 2). The object velocity as a function of time is determined by dividing the change in object position between consecutive frames by the time in between frames. The object was a plastic sphere with a mass *m*_*b*_ of 0.2 g and radius *r*_*b*_ of 3 mm, being shot vertically into the liquids with impact velocities around 100 m/s using a pressurised CO_2_ propulsion system (Smith & Wesson MP40). Two containers were used, each with the same length (22 cm) and depth (22 cm) but with different widths (2.4 and 22 cm). The velocity decay in cornstarch could only be measured in the thin container due to the suspension’s low opacity, where wall effects could influence the measurements. Therefore, the velocity decay in water and PVA were measured in both the small (2.4 cm width) and large (21.6 cm width) containers. For each fluid, twenty to thirty shots were recorded and averaged. The error of the measurements are given by the standard deviation of these averaged shots.

**Figure 3:**
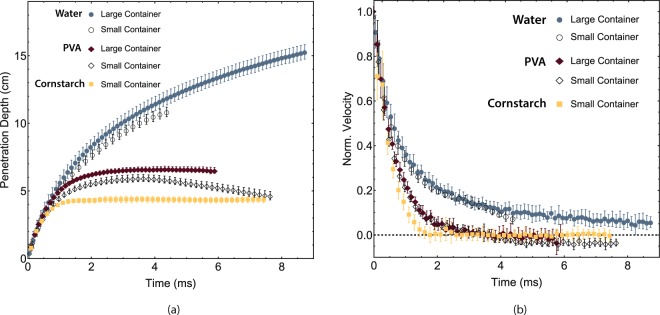
Measured object position (**a**) and normalised velocity (**b**) in large and small containers for demineralised water (large container: blue filled circles; small container: black open circles), cornstarch (small container: yellow squares) and PVA Borax (large container: red filled diamonds; small container: black open diamonds). Here, t = 0 is the moment of impact.

The positions and (normalised) velocity data extracted from the high-speed footage are given in Fig. 3. Figure 3a shows that the object reaches a significantly larger depth in water than in the other fluids. Furthermore, in cornstarch and PVA, the object is completely stopped. This is also evident from the velocity data (Fig. 3b) as the velocity decreases faster in cornstarch and PVA compared to water. These outcomes show that indeed the non-Newtonian characteristics of both the shear thickening and the viscoelastic fluid enhance the object stopping power of the liquid, as the densities are very similar and that the Reynolds number (see below) is high.

The cavity formed during object impact in the large container for water and PVA show similarities with the cavities reported in literature. Aristoff *et al*. investigated the influence of the Weber number $We=\frac{\rho {r}_{b}{v}^{2}}{\sigma }$ and Bond number $Bo=\frac{{\rm{\Delta }}\rho g{r}_{b}^{2}}{\sigma }$ on the cavity formed during object impact^25^. Here *ρ* and *σ* are the density and surface tension of the fluid, *r*_*b*_ and *v* the radius and impact velocity of the bullet and Δ*ρ* and *g* the difference in density between the object and fluid and the gravitational constant, respectively. The Bond number in our study is roughly 0.8, while the Weber is in the order of 10^5^. For water, we observe that the cavity closes at the surface (known as a surface seal) followed by a single pinch off event (see Supplementary Video 1). This is in agreement with the results of Aristoff *et al*. reported in similar *We* and *Bo* regimes^25^. The only difference is that the cavity at high impact velocities collapses into smaller and significantly more bubbles compared to Aristoff *et al*., which is probably due to the higher Weber number reported here. For PVA the cavity shows a similar roughness at the edge of the cavity as reported by Akers *et al*., which is due to the rupture of the viscoelastic fluid^26^. However, instead of a pinch off event we observe that the cavity in PVA expands outwards and subsequently collapses again (see Supplementary Video 2). Interestingly, this behaviour is similar to the temporal shape evolution of cavities in high viscous fluids^27^, with the only exception that no pinch off occurs in PVA and that the object remains inside the cavity.

Comparing the cavity shapes of the small and large containers shows that wall effects have an influence on the cavity shape: For water, the surface seal event observed in the large container does not take place in the small container (see Supplementary Video 3). For PVA, a rebound of the bullet is observed in the small container (see Supplementary Video 4). Figure 3a shows the influence of wall effects on the penetration depth: for both water and PVA the penetration depth is slightly lower in the small container (open black symbols), where the rebound of the object in PVA is also observed (Fig. 2c). Although wall effects seem to influence the penetration depth, the effects seem negligible in the velocity decay in both water and PVA (Fig. 3b), with the exception being the negative velocity in the PVA small container due to the rebound. Using these results, we conclude the velocity decay in water and PVA is not influenced by wall effects, allowing us to compare the measurements of water and PVA in the large container with the measurements of cornstarch in the small container.

It is surprising that the velocity decay in cornstarch and PVA is similar at high impact velocities but that for low velocities the object oscillate inside viscoelastic fluids^26^, while the object impact cornstarch slow down significantly and sinks down afterwards^13^. It is possible that it depends on the strain imposed on the fluid: For low strains, it is possible that it is more difficult for the object to move through viscoelastic fluids than through shear thickening fluids, which might be explained by the big difference of *G*′ and *G*″ for cornstarch and PVA. At higher velocities this difference decreases significantly and the storage and loss modulus of PVA and cornstarch become comparable.

## Velocity Decay Models

In order to determine how the bullet’s kinetic energy changes from the data presented above, we use models describing the velocity decay in Newtonian, viscoelastic and shear thickening fluids.

### Inertial model

For a Newtonian fluid, the force *F*_*D*_ counteracting on the object depends on the Reynolds number, which is the ratio between the inertial and viscous forces of the system:

1 $$Re=\frac{{\rm{inertial}}\,{\rm{forces}}}{{\rm{viscous}}\,{\rm{forces}}}=\frac{\rho {r}_{b}v}{\eta },$$

where *ρ* is the density of the fluid, *r*_*b*_ the radius of the object (and hence the characteristic length scale of our system), *η* the viscosity and *v* the impact velocity of the object. For small *Re* ($Re\ll 1$), the viscous forces dominate. In this regime, *F*_*D*_ is given by the Stokes drag equation for a spherical object^6^:

2 $${F}_{D}=6\pi \eta {r}_{b}\frac{dz(t)}{dt},$$

where *z*(*t*) is the position of the object at time *t* (with *z*(0) = 0 describes the moment of impact). For large *Re* ($Re\gg 1$), the inertial forces dominate and *F*_*D*_ can be approximated with the drag equation:

3 $${F}_{D}=-\,{C}_{d}\pi \rho {r}_{b}^{2}\,{(\frac{dz(t)}{dt})}^{2},$$

with *C*_*d*_ defined as the hydrodynamic drag coefficient of the object. Using *r*_*b*_ = 3 mm and *v* = 100 m/s, we obtain a Reynolds number during impact on the order of 10^5^ for water, which is indeed in the large-*Re* regime. To determine the *Re* regime of cornstarch and PVA the complex viscosity η^*^ is used^20^:

4 $${\eta }^{\ast }=\frac{1}{\omega }\sqrt{{(G^{\prime} )}^{2}+{(G^{\prime\prime} )}^{2}}$$

Where *ω* is the angular frequency used in the rheometer measurements. Assuming that the object impact generates a high strain on the liquid during impact, the Reynolds numbers of cornstarch and PVA can be calculated. Using the storage and loss modulus (Fig. 1) for strains larger than 500%, the Reynolds number for both cornstarch and PVA are on the order of 10. Although the Reynolds number of both fluids are lower compared to water, the inertial forces still dominate the viscous forces during impact. Therefore, the drag should be inertial and not viscous (Eq. (3)). Using Newton’s second law, one can then obtain a differential equation for the velocity of the object, using $v\equiv \frac{dz(t)}{dt}$:

5 $${m}_{b}\frac{dv}{dt}=-\,{C}_{d}\pi \rho {r}_{b}^{2}{v}^{2},$$

with *m*_*b*_ the mass of the object. The solution for the impact velocity follows as:

6 $$v=\frac{{v}_{0}}{1+t/{\tau }_{in}},$$

where *v*_0_ is the impact velocity and *τ*_*in*_ an inertial velocity decay time. The theoretical value of the inertial velocity decay time is defined as ${\tau }_{in}=\frac{{m}_{b}}{{C}_{d}\pi {r}_{b}^{2}{v}_{0}\rho }$. This model will be referred to as the inertial model.

### Viscoelastic model

To model a viscoelastic fluid, it is important to note that the fluid can show both viscous and/or elastic behaviour depending on the timescale of the impact^20^. One of the simplest and best-known models that describes viscoelasticity is the Maxwell model^28,29^, in which the viscous and elastic responses of a viscoelastic fluid are approximated with a damper (dashpot) and spring connected in series. The dashpot, with viscosity *η*, describes the viscous behaviour while the spring, with elasticity *E*, describes the elastic behaviour of the fluid. In this model, it is assumed that the dashpot and spring behave according to Newton’s and Hooke’s law, respectively, meaning that both *η* and *E* are independent of the magnitude of the introduced stress. A further assumption is that both the elastic and viscous responses are uncorrelated. Using the Maxwell model, the material response on the impact can be described with the differential equation of a damped harmonic oscillator:

7 $$\frac{{d}^{2}z(t)}{d{t}^{2}}+2{\omega }_{0}\zeta \frac{dz(t)}{dt}+{\omega }_{0}^{2}z(t)=\mathrm{0,}$$

Where ${\omega }_{0}\equiv \sqrt{\frac{E}{{m}_{b}}}$ is defined as the undamped angular frequency of the oscillator and $\zeta \equiv \eta \mathrm{/2}\sqrt{{m}_{b}E}$ the damping ratio. Depending on the elasticity and viscosity of the Maxwell model, the response to an object impact can either be underdamped (*ζ* < 1) or overdamped (*ζ* > 1). If the system is underdamped, the bullet would oscillate within the fluid with a decrease in amplitude over time. However, as the bullet is completely stopped in both cornstarch and PVA (Fig. 3b) without oscillating, we assume that the system is overdamped and the viscous dissipation of the dashpot dominates. With this assumption, a solution of Eq. 7 can be found where the velocity decays exponentially with time:

8 $$v(t)={v}_{0}{{\rm{e}}}^{-\gamma t}$$

Where $\gamma \equiv \frac{\eta }{2{m}_{b}}$ and *v*_0_ is (again) the impact velocity of the object.

Goff *et al*. studied object impact on foams^30^ by using an adaptation of the Kelvin-Voigt model (a model where the spring and dashpot are connected in parallel instead of in series)^20^ where the plasticity of the foam was taken into account^31^. In contrast with Goff *et al*., plasticity does not play a role in our experiments as the fluids are not irreversibly deformed during impact. However, whether the viscous and elastic responses in the model are correlated or not might have an influence on the velocity decay in viscoelastic fluids at low speeds. For example, Akers *et al*. showed that in low velocity impacts (~2.43 m/s) on viscoelastic micellar fluids, the object oscillates. This can be described as a damped oscillation, but how the oscillations change over time is dependent on whether the elastic and viscous responses are correlated (Kelvin-Voigt model) or not (Maxwell model). However, as both cases predict an exponential decay for the velocity at high impact velocities, this is beyond the scope of our paper.

### Added Mass model

Finally, we used the added mass model given by Waitukaitis and Jaeger^13^ to describe the shear thickening response of a suspension on an impacting object. In this model, the response on the external stress is approximated as an inelastic collision in which, as mentioned in the introduction, the objects pushes the suspended particles together to form a solidified plug. This plug pulls the surrounding liquid downward like a ‘snowplough’^13^, adding mass over time that the object needs to displace. Using Newton’s first law, one can obtain the following second-order differential equation:

9 $$({m}_{b}+{m}_{a}(t))\,\frac{{d}^{2}z(t)}{d{t}^{2}}=-\,(\frac{d{m}_{a}(t)}{dt}\frac{dz(t)}{dt})-{F}_{ext},$$

where *m*_*a*_(*t*) equals the increasing mass over time and *F*_*ext*_ an external force which, in our case, is the gravitational force. Then, following the same logic as Waitukaitis *et al*., the added mass over time can be approximated with a cone-like region around the impacting object, increasing with time:

10 $${m}_{a}(t)=\frac{1}{3}\pi \rho C{({r}_{b}+kz(t))}^{2}kz(t),$$

with *r*_*b*_ the radius of the impacting object with a circular surface area. *C* and *k* are defined as the “added-mass coefficient” and “front coefficient”, respectively (for details, see the paper of Waitukaitis and Jaeger^13^). The former is introduced to compensate for the fact that the impacted medium is a liquid instead of a solid, and the latter as the distance between the solidification front of the added mass and the object’s position^13^.

It is important to note that Waitukaitis and Jaeger only tested this model for a limited range of low impact velocities (0.2 < *v* < 2 m/s), where the object only deforms the surface rather than penetrating the material^13^. At high impact velocities, the object penetrates the liquid and creates an air cavity (Fig. 2b). The added mass model does not take the detailed form of the interface into account, and quantitative discrepancies may therefore occur.

## Model Fitting

In order to determine which model describes the experimental data best, we have fitted them to the data by considering the remaining material parameter(s) in the model equations for *v*(*t*) as fitting parameters. Fitting the three models to the data was done using a least squares method.

Since the added mass model does not have an analytical solution, we numerically solved (9) for a predetermined set of fitting parameters *C* and *k* (${10}^{-6}\leqslant C\leqslant 3\cdot {10}^{-5}$ and $0\leqslant k\leqslant 13$). For each combination of *C* and *k*, the sum of square residuals was calculated and the best fit values of *C* and *k* were determined from the combination with the smallest sum of square residuals.

**Figure 4:**
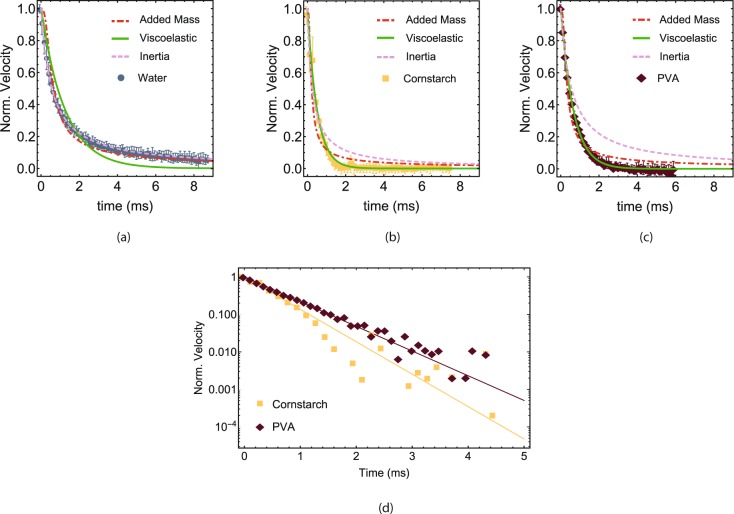
Best fits of the added-mass (red), viscoelastic (green) and inertial (blue) models on the average data for water (**a**), cornstarch (**b**) and PVA (**c**). (**d**) Comparison of the average data and the best overdamped viscoelastic model fits of cornstarch and PVA. For reasons of clarity, the uncertainty of the measurements have not been plotted.

The best fit of each model, together with the averaged data, are shown in Fig. 4. As the Reynolds number of water is in the order of 10^5^, the expectation is that the inertial model gives the best fit on the data, which is indeed shown in Fig. 4a (*R*^2^ = 0.999). The added mass model also gives a relatively good fit (*R*^2^ = 0.97). However, this result is unphysical as the object cannot create a solidified plug as water does not contain suspended particles. Therefore, the good fit of the added mass model for water is unphysical. For the inertial model, the theoretical value of the inertial velocity decay time *τ*_*in*_ of water can be calculated using the mass and radius of the object, the density of water ($997.0\,\frac{{\rm{kg}}}{{{\rm{m}}}^{3}}$)^32^, the impact velocity and *C*_*d*_. As the Reynolds number for bullet impact in water is roughly 3.3 ⋅ 10^5^, the hydrodynamic drag coefficient has a value of around 0.13^33,34^. Using these numbers, the theoretical value of *τ*_*in*_ is equal to 0.54 ms^−1^, which is nearly identical to the best fit value of *τ*_*in*_ (0.563 ± 0.004 ms^−1^) found for water. The good agreement between the theoretical and best fit value of *τ*_*in*_ suggest that the velocity decay of water can be predicted from hydrodynamic theory.

Several studies state that the fluid’s displacement by the object has to be accounted for during low velocity impact in Newtonian fluids^35–37^. This is done by adding an mass term equal to $\frac{1}{2}\rho {V}_{b}$ to Eq. (5), with *V*_*b*_ the volume of the impacting object. Due to the small radius of the impacting object, the mass of the displaced fluid is around 10% for the object’s mass, which is relatively small and could be negligible. This is confirmed by the good fit of the inertial model on the measured velocity decay in water.

More interestingly, the temporal velocity decay in cornstarch (Fig. 4b) is described best by an overdamped viscoelastic model (*R*^2^ = 0.96) while the added mass model poorly fits to the data (*R*^2^ = 0.86). For PVA (Fig. 4c), the temporal velocity decay is also best described by the overdamped viscoelastic model (*R*^2^ = 0.997). For the added mass model, Waitukaitis and Jaeger state that the surface depression forms due to the fact that the fluid has to be globally incompressible^13^. It could be that the fast deformation of the fluids at high impact velocities violates this assumption and could be the reason why the added mass model does not work for high velocity impact. However, with the presented data a definite conclusion cannot be given.

To compare the velocity decay of cornstarch and PVA, the data and corresponding best fit of the overdamped viscoelastic model are plotted in Fig. 4d. The log plot show the overdamped viscoelastic model as a straight line, where the best fit value of *γ* gives the slope of the line. Figure 4d shows that the velcocity decay in cornstarch (*γ*_*c*_ = 1.99 ± 0.08) is higher than that for PVA (*γ*_*p*_ = 1.56 ± 0.02), which is only possible if the complex viscosity of cornstarch starts to surpass that of PVA at high strains (using Eq. (4) and Fig. 1).

Our results suggest that the enhanced bullet stopping power in both cornstarch (a suspension) and PVA (a weakly cross-linked polymer gel) is determined by the viscoelastic properties of the material. These findings indicate that, contrary to the established beliefs^7–12^, the velocity decay of an object impacting at high velocities on a shear thickening material is not determined by the shear thickening properties of the fluid but by its viscoelastic properties.

## Conclusion

Summarising, the main goal of this study was to test whether the stopping power of an object in shear thickening fluids is due to its shear thickening or its viscoelastic properties at high impact velocities. By measuring the velocity decay of a high-speed impacting object in three different types of fluids, we have shown that both the shear thickening fluid cornstarch and viscoelastic fluid PVA have a (mainly) viscoelastic response on the penetrating object. Therefore, the stopping power of a shear thickening liquid depends more on its viscoelastic properties rather than its shear thickening properties, implying that, among other things, the research for better liquid body-armour would benefit from shifting focus from shear thickening to viscoelastic properties of the fluid.

## Supplementary information

Supplementary Information

Supplementary Video 1

Supplementary Video 2

Supplementary Video 3

Supplementary Video 4
