# Supplementary material for: High-velocity impact of solid objects on Non-Newtonian Fluids
Source: Sci Rep. 2019 Feb 4;9:1250. doi: 10.1038/s41598-018-37543-1 (PMC6362045; doi:10.1038/s41598-018-37543-1)
Supplement: Supplementary file 1 — Supplementary Information [file 41598_2018_37543_MOESM1_ESM.pdf]

# High-velocity impact of solid objects on Non-Newtonian Fluids

T. C. de Goede,[1,*] K. G de Bruin,[1,2] and D. Bonn[1,†]

[1] *Van der Waals-Zeeman Institute, Institute of Physics, University of Amsterdam, Science Park 904, 1098 XH Amsterdam, Netherlands*

[2] *Netherlands Forensic Institute, Laan van Ypenburg 6, 2497 GB The Hague, Netherlands*

(Dated: November 28, 2018)

## SUPPLEMENTARY VIDEOS

### Supplementary video 1

High speed video (framerate = 8300 fps) of a spherical object impacting water in the large liquid container (dimensions: length 22cm, depth 22cm and width 22cm).

### Supplementary video 2

High speed video (framerate = 8300 fps) of a spherical object impacting PVA Borax in the large liquid container (dimensions: length 22cm, depth 22cm and width 22cm).

### Supplementary video 3

High speed video (framerate = 4700 fps) of a spherical object impacting on water in the small liquid container (dimensions: length 22cm, depth 22cm and width 2.4cm)

### Supplementary video 4

High speed video (framerate = 6006 fps) of a spherical object impacting on PVA Borax in the small liquid container (dimensions: length 22cm, depth 22cm and width 2.4cm)

* T.CdeGoede@uva.nl

† d.bonn@uva.nl
